# Q Fever in Woolsorters, Belgium

**DOI:** 10.3201/eid1712.101786

**Published:** 2011-12

**Authors:** Pierre Wattiau, Eva Boldisova, Rudolf Toman, Marjan Van Esbroeck, Sophie Quoilin, Samia Hammadi, Hervé Tissot-Dupont, Didier Raoult, Jean-Marie Henkinbrant, Mieke Van Hessche, David Fretin

**Affiliations:** Veterinary and Agrochemical Research Centre, Brussels, Belgium (P. Wattiau, M. Van Hessche, D. Fretin);; Slovak Academy of Sciences, Bratislava, Slovakia (E. Boldisova, R. Toman);; Institute of Tropical Medicine, Antwerp, Belgium (M. Van Esbroeck);; Institute of Public Health, Brussels (S. Quoilin, S. Hammadi);; Université de la Méditerranée, Marseille, France (H. Tissot-Dupont, D. Raoult);; Occupational Health Medicine Group Provikmo, Verviers, Belgium (J.-M. Henkinbrant)

**Keywords:** Q fever, Coxiella burnetii, bacteria, seroprevalence, woolsorters, zoonoses, Belgium

**To the Editor:** Recent outbreaks of Q fever in the Netherlands and the United Kingdom raised public awareness about this ubiquitous bacterial disease known for decades to circulate worldwide (*1**–**4*). The disease, which ranges from a self-recovering influenza-like illness to pneumonia and severe meningoencephalitis, myocarditis, or endocarditis, is usually transmitted from animals to humans by airborne particles derived from contaminated feces and birth products. Clinical symptoms develop in only ≈40% of infected humans (*1*). In ≈1%–2% of these persons, symptoms evolve toward the chronic form of the disease, which can be life-threatening (*5*).

Q fever seroprevalence in the general population in Europe ranges from 2.4% to >30% in some countries in the Mediterranean region (*5*). Despite improved awareness during the past 3 years and geographic proximity with the Netherlands, few human cases were reported in Belgium (14, 27, and 33 cases in 2007, 2008, and 2009, respectively) (*6*). A retrospective survey of blood donors in the Netherlands showed a seroprevalence of 2.4% before the start of outbreaks (*7*). In France, the prevalence of the disease in the Nord-Pas-de-Calais region bordering Belgium is low and accounts for only 0.5% of Q fever cases in France (*8*). Seroprevalence of the general population in Belgium, although unknown, is thus probably comparable with that in neighboring countries in absence of outbreaks and is not expected to exceed 5%.

We report a serologic, epidemiologic, and microbiological Q fever survey conducted in a scouring factory that processed wool and goat hair products in Belgium. No acute Q fever episodes were previously reported by the factory workers. Data on clinical symptoms and risk factors were obtained in face-to-face interviews, and associations with seropositivity were explored by using regression analysis. Airborne dust collected inside the factory during goat hair processing (*9*) contained 10^2^–10^3^ genome equivalents of *Coxiella burnetii*, the Q fever agent, per liter of air as estimated by real-time PCR (**Laboratoire Service International,** Lissieu, France) (Table A1). Sheep wool processing generated less dust and resulted in a *C. burnetii* air load that never reached 10 genome equivalents/L in our analyses. No information is available about the infectivity or viability of air-suspended *C. burnetii* in the studied environment.

Q fever serologic analysis was conducted by using an in-house ELISA for serum samples from 69 workers obtained annually during 2007–2009. Results of samples from the third year were confirmed in parallel by using an immunofluorescent assay (IFA) (Focus Diagnostics, Cypress, CA, USA) in the reference laboratory in Belgium and with follow-up samples in cases of noninterpretable or suspected serologic profiles. The 3-year cumulative seroprevalence was 50.7% (Table). This high value likely results from occupational exposure inside the factory. However, one cannot exclude that characteristics such as traveling abroad, farming, or living near farm animals might account for part of the seroprevalence. Nevertheless, such characteristics could not be associated with positive serologic results in our epidemiologic analysis.

The serologic status of 2 workers (T3 and T42) was compatible with ongoing chronic Q fever as assessed by IFA and ELISA (Table). Another IFA conducted in the reference laboratory in France confirmed a serologic status compatible with chronic Q fever for worker T3 and detected anti–phase I and II IgA in this worker. However, chronic status was not confirmed for worker T42 by this laboratory (Table A1).

Profiles of other workers in the cohort were characterized by increased anti–phase II IgG, IgM, or both (9/69, 13%) over a 2-year period. These profiles suggest relapse and may result from continuous exposure to the Q fever agent, which led to reinfection or repeated stimulation of the immune response. Molecular testing did not detect *C. burnetii* DNA in any blood sample, and clinical examinations did not detect endocarditis in worker T3 as analyzed by positron emission tomography and transthoracic and transesophagian echocardiography. However, infection of tissues other than the heart in this worker cannot be ruled out.

Our results indicate high seroprevalence of Q fever among workers at the scouring factory studied. Continuous exposure to the Q fever agent was the likely cause of atypical antibody responses evoking a chronic or relapsing disease in the absence of any clinical symptom. These results indicated the need to analyze paired serum samples and to rely on medical follow-up before establishing a definitive diagnosis.

Given the continuous occupational risk to which these workers are exposed, hiring of pregnant women or persons with underlying medical conditions, such as valvulopathy or immunologic depression, should be avoided. Moreover, annual serologic testing should be conducted on all exposed persons to detect any evolution toward the chronic form of the disease, which can be life-threatening. Although less dangerous than anthrax, Q fever is still a highly prevalent occupational disease that affects persons working with animal hairs in industrial environments and commonly referred to as woolsorters (*10*).

**Table:** Serologic results for Q fever in woolsorters, Belgium, 2007–2009*

Serologic status	ELISA† (years 1–3)	IFA‡ (year 3)	Confirmed§ (years 1–3)
No. negative	29	27	34
No. nonspecifically reactive	NA	9	NA
No. with past infection	31	31	26
No. with recent or active infection	7	NA	8
No. with chronic infection	2	2	1
% Seroreactive	57.9	47.8	50.7

*IFA, immunofluorescent assay; NA, not applicable. †Conducted on samples collected annually for 3 y. Serologic titer for chronic Q fever: phase I IgG >12,800 and > phase II IgG; for recent or active infection, phase II IgG >1,600 and phase II IgM >800; for past infections, phase II IgG >1,600 and phase II IgM <800. ‡Conducted on paired samples in cases of suspected or noninterpretable initial results; conducted on single samples in all other cases. Serologic status was defined at year 3 according to the instructions of the test kit manufacturer (Focus Diagnostics, Cypress, CA, USA). §Workers with test results above the threshold at least once over a 3-y period by ELISA and IFA. Serologic status was adjusted on the basis of the 3-y projection and retesting in the reference laboratory in France.

## Appendix

**Table A1:** *Coxiella burnetii* TaqVet quantitative real-time PCR kit results, Belgium, 2007–2009*

Material processed	Origin	Batch no.	Result	C_t_	*C. burnetii* concentration, GenEq/mg
Goat hair	Afghanistan and Russia	07/13528 1.1A	+	30.0	10^3^–10^4^
Goat hair	Afghanistan and Russia	07/13528 1.1B	+	29.0	10^3^10^4^
Goat hair	Afghanistan and Russia	07/13528 1.1C	+	28.5	10^3^–10^4^
Goat hair	Afghanistan	06/11664.5	+	27.0	10^3^–10^4^
Goat hair	Asia	08/703.4	+	27.0	10^3^–10^4^
Goat hair	Asia	08/703.4 4B	+	28.5	10^3^–10^4^
Goat hair	Asia	08/703.4 4C	+	27.0	10^3^–10^4^
Wool	The Netherlands	33945	+	34.0	<10^2^
Wool	France and the Netherlands	33961	+	34.0	<10^2^
Wool	Italy	33944	+	36.0	<10^2^
Wool	Hungary	33954	–	>40	<10
Wool	France and Switzerland	33935	+	36.0	<10^2^

*DNA was extracted by using the QIAamp DNA Stool Mini Kit (QIAGEN, Valencia, CA, USA) from 20-mg of airborne dust samples suspended in 0.5 mL of MiliQ water (Millipore, Billerica, MA, USA). PCR (Laboratoire Service International, Lissieu, France) was conducted according to the manufacturer’s instructions. C_t_, cycle threshold; GenEq, genome equivalents; +, positive; –, negative.
